# Association of Paternal Workplace and Community Social Capital With Paternal Postnatal Depression and Anxiety: A Prospective Study

**DOI:** 10.3389/fpsyt.2022.782939

**Published:** 2022-02-17

**Authors:** Satomi Doi, Aya Isumi, Takeo Fujiwara

**Affiliations:** ^1^Department of Global Health Promotion, Tokyo Medical and Dental University (TMDU), Tokyo, Japan; ^2^Japan Society for the Promotion of Science, Tokyo, Japan

**Keywords:** father, community social capital, workplace social capital, depression, anxiety, postnatal mental health

## Abstract

**Objective:**

The aim of this study is to examine the association between workplace and community social capital and fathers' postpartum depression and anxiety at 3 months after delivery in Japan.

**Methods:**

Fathers who had babies delivered in two obstetric clinics in Tokyo, Japan were recruited to take part in the study (response rate = 76.2%). Participants completed questionnaires measuring workplace and community social capital, depression, and anxiety at 1 week and a follow-up at 3 months post-delivery (N = 398). Multiple linear regression analyses were performed with multiple imputation for missing data (at most, N = 60, 15.1%).

**Results:**

Community social capital was inversely associated with both depressive symptoms (β = −0.21, 95%CI = −0.33 to −0.08) and anxiety (β = −0.38, 95%CI = −0.66 to −0.11) at 3 months, after adjusting for covariates. No association was found between workplace social capital and depressive symptoms and anxiety.

**Conclusion:**

Paternal community social capital, but not workplace social capital, was shown to be a preventive factor for paternal depression and anxiety up to 3 months post-delivery. To prevent paternal mental health problems during the postpartum period, an intervention to promote paternal community, rather than workplace, social capital may be warranted.

## Introduction

Paternal mental health problems, such as depression and anxiety during the perinatal period, are common issues (1, 2) presenting a public health concern, in addition to maternal perinatal mental health. Previous studies estimated that 8.4% of fathers across North and South America, the United Kingdom, Asia, and Australia/Oceania (3, 4), and 8.8% of those in Japan (5) showed paternal depression within 1-year postnatal. Postnatal paternal depression is associated with poor satisfaction with the marital relationship (6, 7), increased maternal depression (8), poor father-infant interaction (9, 10), poor child development (10), and increased child maltreatment (5). Furthermore, a previous study found that paternal postnatal depression is associated with depression in offspring (only female) at 18 years of age, which was mediated by maternal depression (11).

In terms of paternal anxiety, systematic reviews found that from 2.4 to 51.0% of fathers show some anxiety disorders during the postnatal period (12, 13). Paternal anxiety, which may raise the risk of paternal depression (14), is associated with poor paternal parenting self-efficacy (15) and poor father-infant interaction (16). A previous study suggests that, in addition to treatment for depression, assessment and treatment of paternal anxiety are needed (13).

In order to prevent paternal postnatal depression and anxiety, preventive factors must be identified. In general, it is well-established that social capital plays an important role in preventing depression and anxiety (17–19). Social capital is defined as resources that are available *via* civic participation in voluntary organizations, norms of mutual aid and reciprocity, and a level of interpersonal trust (20). Though there is still debate regarding its precise definition, social capital can be categorized as individual level and contextual level, such as community, school, or workplace (21). Perceived social support can be considered as one aspect of individual level social capital (22, 23). For example, people with a high individual level of social capital are more likely to have access to psychosocial resources to cope with their mental distress, considered as perceived social support (24). Further, social capital induces structural aspects, such as belonging to a group (25), which improve mental health (26). Among mothers during the postnatal period, a higher level of social capital was associated with lower levels of depression (27–29). However, little is known about the association between social capital and paternal depression and anxiety during the postnatal period.

For fathers, not only community social capital but also workplace social capital may play an important role in preventing depression and anxiety because, compared to mothers, fathers tend to continue working. In this study, community social capital represents trust in neighbors and reciprocity in the neighborhood. The association of community social capital on mental health is well-established (30). Besides the neighborhood, the workplace is considered a major social organization, in which there is both formal and informal face-to-face communication and many sources of social capital (31). Especially in Japan, fathers are less likely to take paternity leave (32), although the rate of paternity leave in Japan has increased slowly (3.2% in 2016, 6.2% in 2018, and 12.7% in 2020) (33). Furthermore, it may be helpful to consider an intervention designed to promote paternal social capital to address community and workplace social capital independently.

In previous studies which examined the association between social capital and mental health problems, participants' socio-economic status—including education level, annual household income, and employment—and history of psychiatric disorders were adjusted for as confounders (34). Further, adverse childhood experience is an important confounder because it is associated with both social capital and mental health problems (35). Additionally, covariates regarding delivery such as paramipara, feelings when pregnancy was confirmed, and paternal childcare leave are adjusted for to examine the association among parents (36). As for possible mediators, paternal postpartum depression and anxiety at 1 week after delivery, maternal depressive symptoms and anxiety at 1 week after delivery, and the number of people who can be consulted about parenting were also adjusted for in our analyses. Thus, the aim of this study is to examine the association between workplace and community social capital and postpartum depression and anxiety among fathers in Japan, at 3 months after delivery, adjusted for possible covariates.

## Methods

### Participants

**Figure 1:**
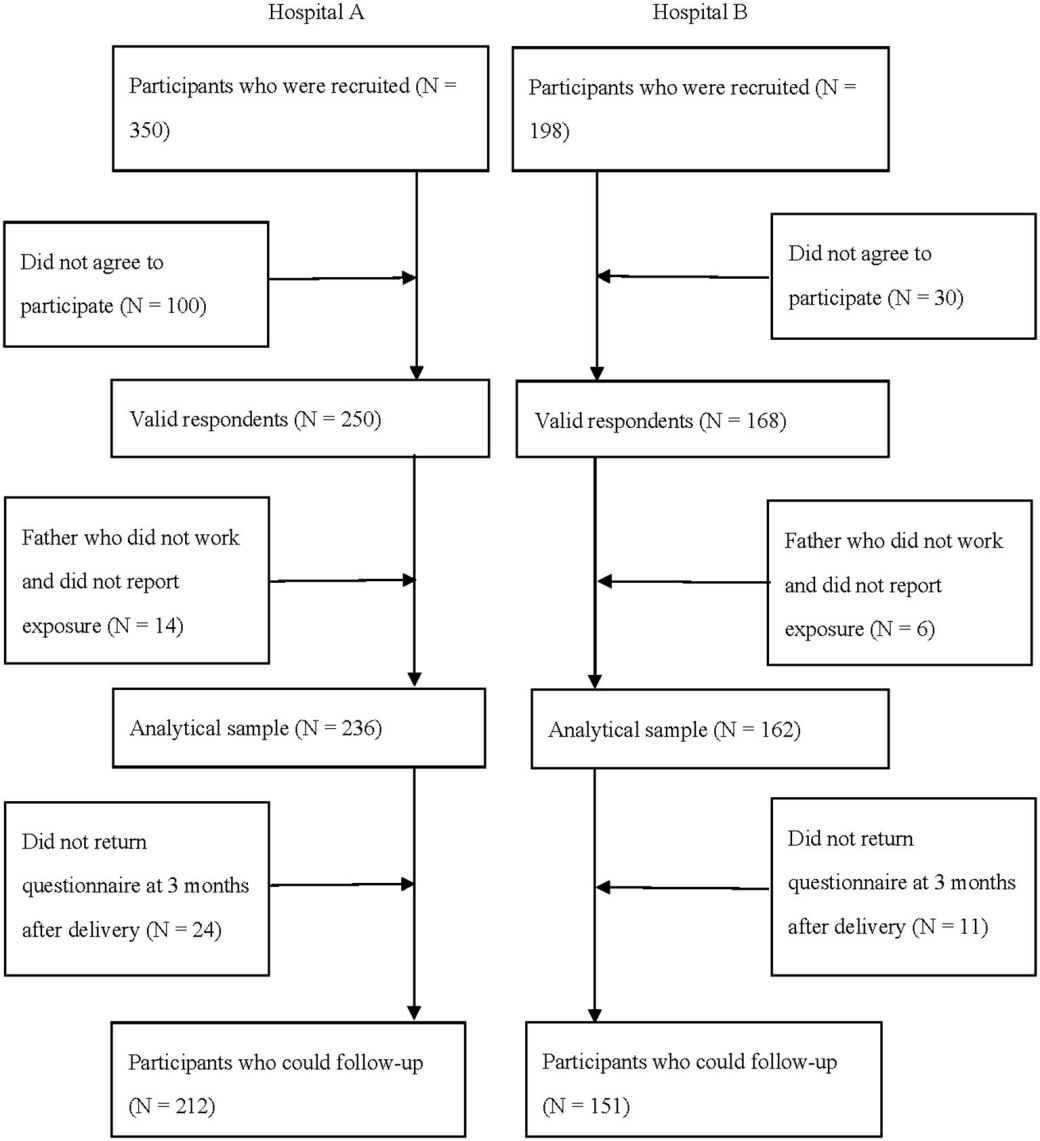
Participant flow chart.

We approached 548 couples who delivered their babies in two obstetrics hospitals in Tokyo, Japan. A total of 350 couples were approached from obstetric hospital A, which is a hospital for high-risk and emergency pregnancies, and 198 couples from hospital B, a general obstetric hospital. During their hospital stay (within 1 week after delivery), the couples completed and returned anonymous questionnaires after written informed consent was acquired. The participants who completed the questionnaire 1 week after delivery included 418 couples (response rate: 76.2%): 250 in hospital A (71.4%) and 168 in hospital B (84.8%). In this study, we excluded fathers who did not work and did not report exposure (i.e., workplace and community social capital) (N = 20); thus, the analytic sample totaled 398 couples. These couples then received follow-up questionnaires *via* mail 3 months after delivery, and 363 questionnaires of the analytical sample were returned completed (follow-up rate: 91.2%): 212 from hospital A (89.8%) and 151 from hospital B (93.2%) (Figure 1). As missing data at 3 months after delivery were imputed using multiple imputations, the analytical sample was 398 couples.

### Measures

Community and workplace social capital were assessed via the father's questionnaire when the baby and the mother were discharged from the hospital (1 week after delivery). Community social capital included the following four questions, used in previous studies (37–39), rated on a scale of 1 to 5: (1) “Do you think that people in your neighborhood can be trusted?”; (2) “Do you think that people in your neighborhood are willing to help their neighbors?”; (3) “Do you think that people in your neighborhood trust each other?”; (4) “Do you think that people in your neighborhood help each other?” A higher total score, ranging from 4 to 20, denotes a higher level of community social capital. Cronbach's α in this study was 0.89. Workplace social capital was assessed using the Japanese version of the workplace social capital scale (40), which was developed from the original version (41). The scale has eight items (e.g., “Our supervisor treats us with kindness and consideration,” “People feel understood and accepted by each other,” and “We can trust our supervisor.”), rated on a scale of 1 to 5. The mean score ranges from 1 to 5, where a higher means score denotes a higher level of workplace social capital. The Japanese version scale has good internal consistency and reliability (40). In this study, Cronbach's α was 0.92.

Paternal depression was assessed at 1 week and 3 months after delivery. We used the Japanese version of the Edinburgh Postnatal Depression Scale (EPDS) (42), which is a 10-item self-report questionnaire with a scale of 0 to 3. A higher total score, ranging from 0 to 30, denotes a higher level of depressive symptoms. In this study, the Cronbach's α of depression at 3 months after delivery was 0.80. Paternal anxiety was also assessed at 1 week and 3 months after delivery. We used the Japanese version of the State-Trait Anxiety Inventory (STAI) (43), which is a 40-item (20-item trait and 20-item state anxiety) self-report questionnaire with a scale of 1 (“not at all”) to 4 (“very much so”). In this study, we used the score of state anxiety, where a higher total score, ranging from 20 to 80, denotes a higher level of state anxiety. In this study, the Cronbach's α of anxiety at 3 months after delivery was 0.92.

Regarding covariates, in addition to the obstetric hospital where mothers gave birth, paternal basic demographics, job, history of psychiatric disorders, adverse childhood experiences, history of delivery, paternal feelings when pregnancy was confirmed, maternal mental health, and the number of people who can be consulted about parenting, which were assessed within 1 week after delivery, were included. Fathers were asked about their age, education level (“junior high school,” “high school,” “technical college or junior college,” “college education,” “graduate college,” or “unknown”), employment (“full-time job,” “part-time job,” “self-employed,” homeworker,” or “other”), paternal childcare leave (“yes,” “planning to take,” or “no”)—assessed at 3 months after delivery—history of psychiatric disorders (“yes” or “no”), paternal adverse childhood experiences—including 8-items, such as parental loss, abuse, and neglect from parents—(“yes” or “no”), and feelings when pregnancy was confirmed (“happy,” “unexpected but happy,” “unexpected and confused,” “did not know what to do,” “no feelings,” or “other”). Mothers were asked about annual household income (JPY 2,000,000 or less, JPY 2,010,000–4,000,000, JPY 4,010,000–6,000,000, JPY 6,010,000–8,000,000, JPY 8,010,000–10,000,000, JPY 10,010,000–15,000,000, JPY 15,010,000 or more,” or “unknown”) and history of delivery (“primipara” or “multipara”). Normal pregnancy was assessed as whether the mother had any major complications during the pregnancy using one item: “Did you have a normal pregnancy?” (“yes” or “no”). Maternal depressive symptoms were assessed using the EPDS, and maternal anxiety was assessed using the STAI.

### Ethics

This study was approved by the Institutional Review Board of the National Center for Child Health and Development (730) and Tokyo Medical and Dental University (M2017-078).

### Statistical Analysis

We performed multiple linear regression analyses to examine the associations of community and workplace social capital within 1 week after delivery with paternal depressive symptoms (i.e., EPDS total score) and anxiety (i.e., STAI state total score) at 3 months after delivery. After estimating the crude model, Model 1 added paternal education, age, annual household income, employment, childcare leave, adverse childhood experiences, paternal history of psychiatric disorders, history of delivery, normal pregnancy, and paternal feelings when pregnancy was confirmed. In addition to the covariates in Model 1, Model 2 further added paternal depressive symptoms (only for depressive symptoms as the outcome) or anxiety (only for anxiety as the outcome) at 1 week after delivery and maternal depressive symptoms and anxiety at 1 week after delivery. Finally, Model 3 further added the number of people who can be consulted about parenting and both types of social capital into Model 2. We conducted the power calculation (power = 0.90, alpha = 0.01) to compute sample size because a high number of covariates were included in the final model. We found that a sample of at least 161 subjects is required in our analysis, indicating that our sample size is sufficient.

We performed multiple imputations (100 imputed datasets) based on Rubin's rule (44, 45) to address missing values. Missing data were found in outcomes including paternal EPDS and STAI scores at three months after delivery (N = 35, 8.8%), and in covariates including annual household income (N = 22, 5.5%), paternal childcare leave (N = 60, 15.1%), and the number of people who can be consulted about parenting (N = 28, 7.0%). There were no differences in characteristics, workplace social capital, community social capital, depressive symptoms, and anxiety at 1 week after delivery (data not shown). The following variables were used for the multiple imputation: paternal symptoms of attention-deficit/hyperactivity disorder (ADHD) assessed using the Japanese version of the 6-item Adult ADHD Self-Report Scale (ASRS-J-6) (46), autism traits assessed using the Japanese version of the Autism-Spectrum Quotient (AQ) (47), domestic violence from their partner, belonging to an organization, attendance at delivery, and health condition were measured via responses from the father's questionnaire. In addition, maternal adverse childhood experiences, feelings toward her baby (i.e., mother-infant bonding)—assessed using the Japanese version of Mother-to-Infant Bonding Scale (MIBS-J) (48)—type of housing, and child's sex were measured via the mother's questionnaire and used for multiple imputation. All analyses were conducted using STATA version 15.0 SE.

## Results

The distribution of characteristics among participants is shown in Table 1. Approximately 10% of fathers were <30 years old, 80% were college graduates or higher, 20% experienced some adverse childhood experiences, 90% had a full-time job, 10% took or planned to take childcare leave, 2% had a history of psychiatric disorders, and 80% felt happy when the pregnancy was confirmed. The percentage of low-income households (i.e., JPY 4,000,000 or less) was 9%. Furthermore, about 20% of fathers participated in group activities in the last year before delivery. On average, fathers had nine (SD = 8.1) people with whom they could consult about parenting.

**Table 1:** Characteristics of sample in this study (N = 398).

	**Total (N** **=** **398)**	
	**N or mean**	**% or SD**
**Obstetric hospital**		
A	236	59.6
B	162	40.4
**Paternal age**		
<30	43	10.8
30– <40	252	63.3
40– <50	96	24.1
50+	7	1.8
**Paternal education**		
High school or less	36	9.0
Some college	36	9.0
College or more	324	81.4
Unknown	2	0.5
**Annual household income (JPN yen)**		
≦4,000,000	36	9.0
4,010,000–8,000,000	141	35.4
8,010,000–15,000,000	161	40.5
15,001,000+	38	9.5
Unknown	17	4.3
Missing	5	1.3
**Paternal ACE total score (0–8)**		
0	323	81.2
1	60	15.1
2+	15	3.8
**Paternal employment**		
Full-time job	364	91.5
Part-time job	4	1.0
Self-employed	24	6.0
Other	6	1.5
**Paternal childcare leave**		
Yes or Planning to take	46	11.6
No	292	73.4
Missing	60	15.1
**Paternal history of psychiatric disorders**		
No	390	98.0
Yes	8	2.0
**History of delivery**		
First birth	273	68.6
Multiparity	124	31.2
Missing	1	0.3
**Normal pregnancy**		
Yes	327	82.2
No	69	17.3
Missing	2	0.5
**Paternal feelings when pregnancy was confirmed**		
Happy	334	83.9
Unexpected but happy	48	12.1
Unexpected and confused/did not know what to do/no feelings/other	13	3.3
Missing	3	0.8
**Social participation in the last year before delivery**		
Yes	74	18.6
No	324	81.4
**Number of people who can be consulted about parenting**	9.47	8.10

Table 2 shows the distribution of paternal workplace and community social capital at 1 week after delivery, and EPDS and STAI scores at 1 week and 3 months after delivery. The paternal workplace and community social capital means were 3.70 (SD = 0.80) and 13.30 (SD = 2.82), respectively. The EPDS score means were 3.11 (SD = 3.17) at 1 week after delivery and 3.92 (SD = 3.77) at three months after delivery. The STAI score means were 34.12 (SD = 8.68) at 1 week after delivery and 34.49 (SD = 8.89) at 3 months after delivery. We also show the correlations among workplace social capital, community social capital, social participation in the last year before delivery, and number of people who can be consulted about parenting in Supplementary Table 1. A significant but weak correlation was found between workplace social capital and community social capital (r = 0.17, *p* < 0.01).

**Table 2:** Distribution of exposure and outcome.

**Variables**	**N**	**Mean**	**SD**	**Minimum**	**Max**
Workplace social capital	398	3.70	0.80	1	5
Community social capital	398	13.30	2.82	4	20
Depressive symptoms at 1 week after delivery	398	3.11	3.17	0	22
Depressive symptoms at 3 months after delivery	363	3.92	3.77	0	22
Anxiety at 1 week after delivery	398	34.12	8.68	20	68
Anxiety at 3 months after delivery	363	34.49	8.89	19	70

Tables 3, 4 shows the results of the linear regression analyses examining the association of social capital with depressive symptoms and anxiety in fathers. In terms of depressive symptoms at 3 months after delivery, community social capital was inversely associated with depressive symptoms (β = −0.21, 95%CI = −0.33 to −0.08) after adjusting for covariates and workplace social capital. Despite workplace social capital being inversely associated with depressive symptoms in Model 1 (β = −0.63, 95%CI = −1.13 to −0.14), the coefficient became non-significant after adjusting for paternal depressive symptoms and maternal mental health problems at 1 week after delivery. Community social capital, paternal part-time job, and paternal depressive symptoms at 1 week after delivery showed significant coefficients among covariates. In terms of anxiety at 3 months after delivery, community social capital was inversely associated with anxiety (β = −0.38, 95%CI = −0.66 to −0.11) at 3 months in Model 3. However, the association between workplace social capital and anxiety remained non-significant after adjusting for paternal anxiety and maternal mental health problems at 1 week after delivery. Community social capital, annual household income (JPY 4,010,000–6,000,000) and anxiety at 1 week after delivery showed significant coefficients among covariates.

**Table 3:** Association between paternal social capital and paternal depressive symptoms at 3 months after delivery after multiple imputations.

		**Workplace social capital**			**Community social capital**			**Model 3 β (95%CI)**
		**Crude**	**Model 1**	**Model 2**	**Crude**	**Model 1**	**Model 2**	
		**β (95%CI)**	**β (95%CI)**	**β (95%CI)**	**β (95%CI)**	**β (95%CI)**	**β (95%CI)**	
Workplace social capital		**−0.71** **(−1.17 to** **−0.24)**	**−0.63** **(−1.13 to** **−0.14)**	−0.15 (−0.60 to 0.30)				−0.01 (−0.47 to 0.45)
Community social capital					**−0.25** **(−0.38 to** **−0.12)**	**−0.26** **(−0.40 to** **−0.12)**	**−0.22** **(−0.34 to** **−0.09)**	**−0.21** **(−0.33 to** **−0.08)**
Obstetrics hospital	A		Ref	Ref		Ref	Ref	Ref
	B		−0.58 (−1.45 to 0.29)	0.07 (−0.72 to 0.86)		−0.55 (−1.41 to 0.32)	0.11 (−0.67 to 0.88)	0.11 (−0.66 to 0.89)
Paternal age			−0.06 (−3.07 to 0.57)	−0.03 (−0.10 to 0.04)		−0.37 (−0.11 to 0.04)	−0.02 (−0.08 to 0.05)	−0.02 (−0.09 to 0.05)
Paternal education	High school or less		0.05 (−1.35 to 1.45)	−0.14 (−1.39 to 1.11)		Ref	Ref	Ref
	Some college		−1.20 (−2.59 to 0.19)	**−1.37** **(−2.60 to** **−0.14)**		−1.63 (−3.44 to 0.18)	−1.48 (−3.08 to 0.13)	−1.41 (−3.03 to 0.20)
	College or more		Ref	Ref		Ref	Ref	Ref
	Unknown		−1.84 (−8.03 to 4.35)	−0.42 (−6.09 to 5.26)		−1.17 (−7.44 to 5.10)	0.50 (−5.22 to 6.22)	0.41 (−5.33 to 6.15)
Annual household income (JPN yen)	≦2,000,000		6.30 (−1.92 to 14.51)	3.00 (−4.22 to 10.22)		Ref	Ref	Ref
	2,010,000–4,000,000		**2.16** **(0.51 to 3.81)**	**1.48** **(0.0002 to 2.95)**		−4.83 (−13.11 to 3.45)	−2.15 (−9.35 to 5.05)	−2.11 (−9.32 to 5.10)
	4,010,000–6,000,000		**1.46** **(0.17 to 2.75)**	0.84 (−0.31 to 1.99)		−5.32 (−13.49 to 2.86)	−2.60 (−9.72 to 4.51)	−2.54 (−9.67 to 4.59)
	6,010,000–8,000,000		Ref	Ref		Ref	Ref	Ref
	8,010,000–10,000,000		**1.32** **(0.03 to 2.60)**	**1.34** **(0.19 to 2.49)**		−5.61 (−13.84 to 2.63)	−2.10 (−9.27 to 5.07)	−2.07 (−9.24 to 5.11)
	10,000,000–15,000,000		**1.45** **(0.25 to 2.65)**	**1.42** **(0.35 to 2.49)**		−5.67 (−13.89 to 2.56)	−2.15 (−9.31 to 5.00)	−2.10 (−9.26 to 5.07)
	15,001,000+		0.35 (−1.18 to 1.88)	0.56 (−0.82 to 1.93)		−6.81 (−15.05 to 1.22)	−3.13 (−10.31 to 4.05)	−3.07 (−10.26 to 4.13)
Paternal ACE total score	0		Ref	Ref		Ref	Ref	Ref
	1		0.31 (−0.77 to 1.49)	0.38 (−0.58 to 1.35)		0.32 (−0.75 to 1.40)	0.38 (−0.57 to 1.33)	0.38 (−0.57 to 1.33)
	2+		1.24 (−0.79 to 3.27)	0.30 (−1.51 to 2.11)		1.21 (−0.80 to 3.22)	0.14 (−1.65 to 1.92)	0.11 (−1.68 to 1.90)
Paternal employment	Full-time job		Ref	Ref		Ref	Ref	Ref
	Part-time job		**5.21** **(1.47 to 8.94)**	**4.83** **(1.50 to 8.16)**		**5.17** **(1.47 to 8.87)**	**4.74** **(1.46 to 8.02)**	**4.68** **(1.40 to 7.97)**
	Self-employed		0.65 (−1.02 to 2.32)	0.44 (−1.05 to 1.92)		0.40 (−1.24 to 2.03)	0.45 (−0.99 to 1.89)	0.43 (−1.05 to 1.91)
	Other		−0.32 (−3.95 to 3.32)	0.69 (−2.59 to 3.96)		−0.15 (−3.77 to 3.46)	0.96 (−2.28 to 4.19)	0.99 (−2.25 to 4.23)
Paternal childcare leave	Yes or Planning to take		Ref	Ref		Ref	Ref	Ref
	No		−0.52 (−1.71 to 0.66)	−0.46 (−1.52 to 0.60)		−0.72 (−189 to 0.45)	−0.56 (−1.60 to 0.48)	−0.51 (−1.55 to 0.54)
Paternal history of psychiatric disorders	No		Ref	Ref		Ref	Ref	Ref
	Yes		2.02 (−0.94 to 4.97)	−1.53 (−4.24 to 1.19)		2.21 (−0.70 to 5.13)	–1.61 (−4.28 to 1.07)	−1.63 (−4.31 to 1.06)
History of delivery	First birth		Ref	Ref		Ref	Ref	Ref
	Multiparity		0.11 (−0.73 to 0.94)	−0.04 (−0.96 to 0.88)		0.38 (−0.47 to 1.22)	0.55 (−0.22 to 1.32)	0.51 (−0.27 to 1.28)
Normal pregnancy	Yes		Ref	Ref		Ref	Ref	Ref
	No		−0.11 (−1.13 to 0.91)	−0.04 (−0.96 to 0.88)		−0.04 (−1.04 to 0.98)	−0.06 (−0.96 to 0.85)	−0.05 (−0.96 to 0.86)
Paternal feelings when pregnancy was confirmed	Happy		Ref	Ref		Ref	Ref	Ref
	Unexpected but happy/unexpected and confused/did not know what to do/no feelings/other		0.69 (−0.39 to 1.78)	0.39 (−0.58 to 1.36)		0.71 (−1.89 to 0.45)	0.38 (−0.58 to 1.33)	0.39 (−0.57 to 1.35)
Paternal depressive symptoms at 1 week after delivery				**0.60** **(0.48 to 0.72)**			**0.59** **(0.48 to 0.71)**	**0.59** **(0.47 to 0.71)**
Maternal depressive symptoms at 1 week after delivery				0.05 (−0.07 to 0.16)			0.05 (−0.07 to 0.16)	0.04 (−0.07 to 0.16)
Maternal anxiety at 1 week after delivery				−0.01 (−0.06 to 0.04)			−0.01 (−0.06 to 0.04)	−0.01 (−0.06 to 0.04)
Number of people who can be consulted about parenting								−0.02 (−0.07 to 0.03)

*95%CI, 95% Confidence Interval. Boldface means statistical significant (p < 0.05)*.

*Model 1 adjusted for paternal education, paternal age, annual household income, paternal employment, paternal childcare leave, paternal adverse childhood experiences, paternal history of psychiatric disorders, history of delivery, obstetrics hospital, normal pregnancy, and paternal feelings when pregnancy was confirmed*.

*Model 2 added paternal depressive symptoms at 1 week after delivery, maternal depressive symptoms, and anxiety at 1 week after delivery into Model 1*.

*Model 3 included number of people who can be consulted about parenting and both types of social capital into Model 2*.

**Table 4:** Association between paternal social capital and paternal anxiety at 3 months after delivery after multiple imputations.

		**Workplace social capital**			**Community social capital**			**Model 3 β (95%CI)**
		**Crude**	**Model 1**	**Model 2**	**Crude**	**Model 1**	**Model 2**	
		**β (95%CI)**	**β (95%CI)**	**β (95%CI)**	**β (95%CI)**	**β (95%CI)**	**β (95%CI)**	
Workplace social capital		**−3.07** **(−4.14 to** **−2.00)**	**−2.94** **(−4.09 to** **−1.80)**	−0.72 (−1.74 to 0.31)				−0.55 (−1.59 to 0.49)
Community social capital					**−0.49** **(−0.80 to** **−0.17)**	**−0.53** **(−0.86 to** **−0.20)**	**−0.40** **(−0.66 to** **−0.13)**	**−0.38** **(−0.66 to** **−0.11)**
Obstetrics hospital	A		Ref	Ref		Ref	Ref	Ref
	B		**−2.82** **(−4.83 to** **−0.81)**	−0.98 (−2.69 to 0.73)		**−2.82** **(−4.87 to** **−0.76)**	−0.88 (−0.90 to 2.96)	−0.91 (−2.61 to 0.79)
Paternal age			−0.02 (−0.20 to 0.15)	−0.04 (−0.18 to 0.11)		0.03 (−0.14 to 0.21)	−0.01 (−0.15 to 0.14)	−0.01 (−0.15 to 0.14)
Paternal education	High school or less		−0.85 (−4.09 to 2.38)	−0.21 (−2.91 to 2.50)		−0.31 (−3.60 to 2.99)	−0.16 (−2.84 to 2.51)	−0.22 (−2.91 to 2.48)
	Some college		−0.70 (−3.91 to 2.52)	−0.45 (−3.12 to2.22)		−1.17 (−4.49 to 2.15)	−0.91 (−3.58 to 1.76)	−0.93 (−3.60 to 1.75)
	College or more		Ref	Ref		Ref	Ref	Ref
	Unknown		−4.98 (−18.87 to 8.91)	−2.69 (−14.75 to 9.37)		−3.71 (−17.90 to 10.48)	−1.32 (−13.33 to 10.70)	−1.24 (−13.26 to 10.78)
Annual household income (JPN yen)	≦2,000,000		0.73 (−18.38 to 19.85)	3.38 (−12.21 to 18.96)		2.28 (−17.44 to 22.00)	4.48 (−11.00 to 19.95)	4.41 (−11.08 to 19.90)
	2,010,000–4,000,000		1.97 (−1.85 to 5.80)	0.26 (−2.93 to 3.46)		2.30 (−1.63 to 6.23)	0.22 (−2.96 to 3.40)	0.20 (−2.99 to 3.38)
	4,010,000–6,000,000		**3.53** **(0.54 to 6.54)**	**2.96** **(0.50 to 5.41)**		**4.27** **(1.19 to 7.35)**	**3.27** **(0.82 to 5.72)**	**2.20** **(0.75 to 5.65)**
	6,010,000–8,000,000		Ref	Ref		Ref	Ref	Ref
	8,010,000–10,000,000		2.81 (−0.13 to 5.75)	1.59 (−0.88 to 4.06)		2.69 (−0.34 to 5.71)	1.75 (−0.71 to 4.20)	1.88 (−0.59 to 4.36)
	10,000,000–15,000,000		1.92 (−0.86 to 4.71)	1.29 (−1.01 to 3.58)		1.28 (−1.55 to 4.11)	1.19 (−1.07 to 3.45)	1.34 (−0.95 to 3.63)
	15,001,000+		1.18 (−2.37 to 4.73)	1.43 (−1.52 to 4.37)		0.79 (−2.86 to 4.44)	1.23 (−1.71 to 4.16)	1.27 (−1.67 to 4.20)
Paternal ACE total score	0		Ref	Ref		Ref	Ref	Ref
	1		0.02 (−2.48 to 2.51)	0.38 (−1.70 to 2.46)		0.14 (−2.41 to 2.69)	0.41 (−1.66 to 2.47)	0.37 (−1.70 to 2.43)
	2+		2.67 (−2.00 to 7.35)	−1.89 (−5.85 to 2.06)		3.28 (−1.50 to 8.05)	−2.16 (−6.09 to 4.36)	−2.21 (−6.14 to 1.73)
Paternal employment	Full-time job		Ref	Ref		Ref	Ref	Ref
	Part-time job		3.89 (−4.74 to 12.52)	2.53 (−4.64 to 9.70)		4.18 (−4.65 to 13.00)	2.42 (−4.69 to 9.53)	2.40 (−4.72 to 9.52)
	Self-employed		0.34 (−3.52 to 4.19)	−0.32 (−3.54 to 2.90)		−1.14 (−5.04 to 2.76)	−0.54 (−3.70 to 4.36)	−0.22 (−3.43 to 2.99)
	Other		0.51 (−7.97 to 8.98)	−0.10 (−7.30 to 7.10)		0.21 (−8.42 to 8.84)	0.27 (−6.90 to 4.36)	0.39 (−6.78 to 7.56)
Paternal childcare leave	Yes or Planning to take		Ref	Ref		Ref	Ref	Ref
	No		−0.96 (−3.69 to 1.77)	−1.33 (−3.61 to 0.95)		−1.65 (−4.43 to 1.13)	−1.59 (−3.85 to 0.67)	−1.52 (−3.79 to 0.76)
Paternal history of psychiatric disorders	No		Ref	Ref		Ref	Ref	Ref
	Yes		0.70 (−6.00 to 7.40)	−1.37 (−8.08 to 4.35)		2.30 (−4.48 to 9.09)	−1.27 (−6.90 to 4.36)	−1.57 (−7.26 to 4.11)
History of delivery	First birth		Ref	Ref		Ref	Ref	Ref
	Multiparity		0.48 (−1.46 to 2.43)	0.20 (−1.48 to 1.88)		1.00 (−1.02 to 3.02)	0.59 (−1.11 to 2.29)	0.63 (−1.07 to 2.33)
Normal pregnancy	Yes		Ref	Ref		Ref	Ref	Ref
	No		0.71 (−1.62 to 3.05)	0.95 (−1.01 to 2.91)		1.28 (−1.10 to 3.65)	1.03 (−0.90 to 2.96)	0.89 (−1.05 to 2.84)
Paternal feelings when pregnancy was confirmed	Happy		Ref	Ref		Ref	Ref	Ref
	Unexpected but happy/unexpected and confused/did not know what to do/no feelings/other		1.54 (−0.96 to 4.04)	−0.16 (−2.25 to 1.94)		1.72 (−0.82 to 4.26)	−0.19 (−2.26 to 1.89)	−0.20 (−2.28 to 1.88)
Paternal anxiety at 1 week after delivery				**0.62** **(0.52 to 0.71)**			**0.63** **(0.54 to 0.72)**	**0.61** **(0.52 to 0.71)**
Maternal depressive symptoms at 1 week after delivery				0.06 (−0.19 to 0.32)			0.05 (−0.20 to 0.30)	0.06 (−0.19 to 0.32)
Maternal anxiety at 1 week after delivery				0.01 (−0.09 to 0.12)			0.01 (−0.09 to 2.96)	0.01 (−0.10 to 0.11)
Number of people who can be consulted about parenting								0.02 (−0.09 to 0.12)

*95%CI, 95% Confidence Interval. Boldface means statistical significant (p < 0.05)*.

*Model 1 adjusted for paternal education, paternal age, annual household income, paternal employment, paternal childcare leave, paternal adverse childhood experiences, paternal history of psychiatric disorders, history of delivery, obstetrics hospital, normal pregnancy, and paternal feelings when pregnancy was confirmed*.

*Model 2 added paternal anxiety at 1 week after delivery and maternal depressive symptoms and anxiety at 1 week after delivery into Model 1*.

*Model 3 included number of people who can be consulted about parenting and both types of social capital into Model 2*.

The results of these analyses using complete data were shown in the Supplementary Tables 2, 3. Most of the associations were similar to the data employing multiple imputations, although effect sizes were slightly higher in the complete data analysis.

## Discussion

This study showed that paternal community social capital, but not workplace social capital, was associated with lower levels of paternal depression and anxiety up to three months after delivery. Thus, we suggest that paternal community social capital may be a preventive factor for paternal depression and anxiety, rather than workplace social capital.

This is the first study to examine the impact of paternal community and workplace social capital on postnatal depression and anxiety. Our findings, in which only community but not workplace social capital was associated with paternal postpartum depression and anxiety, are partially consistent with those of previous studies. In terms of community social capital, it was shown to be associated with mental disorders in a prospective study (49) and a review (18). Moreover, a higher level of maternal social capital during pregnancy was associated with a lower EPDS score at 6–8 weeks after delivery (27). This association can be explained as follows. First, community social capital may alleviate paternal concern about bothering the neighbors due to infant crying. In the Japanese context, caregivers are more likely to be concerned about bothering cohabitants such as grandparents and neighbors due to their infant crying (50). Second, fathers with a higher level of community social capital may be able to access information and receive parenting-related care, as mothers with a higher level of community social capital can access better prenatal care and delivery care (51). Further studies to identify the mechanism of association between community social capital and paternal postnatal depression and anxiety should be conducted. Nonetheless, community social capital plays a significant role in preventing postnatal depression and anxiety among not only mothers but also fathers, indicating that promoting community social capital for fathers may be effective in preventing paternal postnatal depression and anxiety.

In terms of workplace social capital, we found no association, although previous studies reported that workers with a lower level of workplace social capital showed the onset of depression in Finland (male: 20%) (52), Germany (male: 74.4%) (53), and Japan (male: 77.6%) (54). This discrepancy can be explained by differences in the target population (i.e., age of bearing a child) and the assessment period (i.e., right after delivery). For workers, assuming the family status is stable, the workplace is considered a major social context (55). Thus, employment status, job stress, working hours, and job insecurity have huge impacts on the mental health of workers (56). Workers with a higher level of workplace social capital are more likely to be able to cope with their stress (57), which may lead to lower levels of mental health problems. However, in the case of changing family status, such as bearing a child, fathers may face further stress related to parenting and the relationship with their partner, in addition to job stress during the transition to fatherhood. Therefore, fathers may not be able to cope with the stress related to fatherhood transition through workplace social capital. The type of support and information fathers receive from community and workplace social capital during the perinatal period needs to be identified.

Though there was no association between workplace social capital and mental health problems, the significant coefficient was shown in Model 1 in which depressive symptoms and anxiety at 1 week after delivery was not adjusted. Paternal depressive symptoms and anxiety at 1 week after delivery are considered not only confounders but also mediators on a time-series basis, which indicates overadjustment. Additionally, participants who reported higher levels of depressive symptoms and anxiety might be more likely to perceive social capital negatively. This negative perception due to mental health problems might influence workplace social capital more strongly than community social capital because people spend more time and have greater social relations at the workplace than the community (58). In the current study, the correlation between workplace and community social capital was small (r = 0.17). Thus, the impact of mental health problems on workplace and community social capital might differ. Despite adjusting for a history of psychiatric disorders in our analysis, further studies to assess paternal depressive symptoms and anxiety before and during pregnancy, and a longitudinal study with a larger sample size that excludes fathers with depressive symptoms and anxiety at the baseline, are needed.

The current study has several limitations. First, our findings are limited in generalizability due to the participant recruitment method and paternal characteristics. Study participants were recruited from two obstetrics hospitals in Tokyo, Japan. Furthermore, we found that the annual household income of our participants was higher than in another study that targeted families living in Tokyo (59). Second, both exposure (i.e., paternal social capital) and outcomes (paternal depression and anxiety) were self-reported, which may lead to common method bias for causal inference on the association between paternal social capital and their mental health problems. Further research needs to be conducted to assess mental health using objective measures such as interviews by professionals. Third, although the follow-up rate in this study was high and multiple imputations were performed, it is possible that fathers with lower levels of social capital and severe mental health problems dropped out of our survey. Fourth, there are unmeasured confounders, such as severe obstetric complications during pregnancy, although we could assess normal pregnancy in a subjective way.

## Conclusions

Our findings indicate that a higher level of paternal community social capital at birth, but not workplace social capital, prevents paternal depression and anxiety at 3 months after delivery. Even though fathers show a similar level of postnatal depression as mothers, they are less likely to have social support compared to mothers (60). To date, there are some programs designed to promote social capital for parents, nearly all of which target mothers (61, 62). To prevent paternal mental health problems during the postnatal period, an intervention to promote paternal community, rather than workplace, social capital may be warranted.

## Data Availability Statement

The raw data supporting the conclusions of this article will be made available by the authors, without undue reservation.

## Ethics Statement

This study was approved by the Institutional Review Board of the National Center for Child Health and Development (730) and Tokyo Medical and Dental University (M2017-078). The patients/participants provided their written informed consent to participate in this study.

## Author Contributions

TF designed the study. TF and AI managed administration of the study, including the ethical review process and provided critical comments on the manuscript related to intellectual content. SD analyzed data and drafted the manuscript. All authors have read and approved the final manuscript.

## Funding

This study was supported by Grants-in-Aid for Scientific Research from the Japan Society for the Promotion of Science (JSPS KAKENHI Grant Numbers 15K12735 and 18K13318), the Mitsubishi Foundation, Grants for Social Welfare Activities, a Research Development Grant for Child Health and Development from the National Center for Child Health and Development, and the Ministry of Health, Labour and Welfare (21DA1004 and 19AA100).

## Conflict of Interest

The authors declare that the research was conducted in the absence of any commercial or financial relationships that could be construed as a potential conflict of interest.

## Publisher's Note

All claims expressed in this article are solely those of the authors and do not necessarily represent those of their affiliated organizations, or those of the publisher, the editors and the reviewers. Any product that may be evaluated in this article, or claim that may be made by its manufacturer, is not guaranteed or endorsed by the publisher.

## References

[1] Bandelow B Michaelis S. Epidemiology of anxiety disorders in the 21st century. Dialogues Clin Neurosci. (2015) 17:327. 10.31887/DCNS.2015.17.3/bbandelow 26487813 PMC4610617

[2] Wynter K Rowe H Fisher J. Common mental disorders in women and men in the first six months after the birth of their first infant: a community study in Victoria, Australia. J Affect Disord. (2013) 151:980–5. 10.1016/j.jad.2013.08.021 24119921

[3] Cameron EE Sedov ID Tomfohr-Madsen LM. Prevalence of paternal depression in pregnancy and the postpartum: an updated meta-analysis. J Affect Disord. (2016) 206:189–203. 10.1016/j.jad.2016.07.044 27475890

[4] Rao WW Zhu XM Zong QQ Zhang Q Hall BJ Ungvari GS. Prevalence of prenatal and postpartum depression in fathers: a comprehensive meta-analysis of observational surveys. J Affect Disord. (2020) 263:491–9. 10.1016/j.jad.2019.10.030 31757623

[5] Takehara K Suto M Kakee N Tachibana Y Mori R. Prenatal and Early postnatal depression and child maltreatment among Japanese fathers. Child Abuse Negl. (2017) 70:231–9. 10.1016/j.chiabu.2017.06.011 28633058

[6] Pinto TM Samorinha C Tendais I Figueiredo B. Depression and paternal adjustment and attitudes during the transition to parenthood. J Reprod Infant Psychol. (2020) 38:281–96. 10.1080/02646838.2019.1652256 31392897

[7] Johansson M Svensson I Stenström U Massoudi P. Depressive symptoms and parental stress in mothers and fathers 25 months after birth. J Child Health Care. (2017) 21:65–73. 10.1177/1367493516679015 29156983

[8] Paulson JF Bazemore SD. Prenatal and postpartum depression in fathers and its association with maternal depression: a meta-analysis. JAMA. (2010) 303:1961–9. 10.1001/jama.2010.605 20483973

[9] Koch S De Pascalis L Vivian F Meurer Renner A Murray L Arteche A. Effects of male postpartum depression on father–infant interaction: the mediating role of face processing. Infant Ment Health J. (2019) 40:263–76. 10.1002/imhj.21769 30720878

[10] Ip P Li TM Chan KL Ting AYY Chan CY Koh YW. Associations of paternal postpartum depressive symptoms and infant development in a chinese longitudinal study. Infant Behav Dev. (2018) 53:81–9. 10.1016/j.infbeh.2018.08.002 30213511

[11] Gutierrez-Galve L Stein A Hanington L Heron J Lewis G O'Farrelly C. Association of maternal and paternal depression in the postnatal period with offspring depression at age 18 years. JAMA Psychiatry. (2019) 76:290–6. 10.1001/jamapsychiatry.2018.3667 30586134 PMC6439819

[12] Philpott LF Savage E FitzGerald S Leahy-Warren P. Anxiety in fathers in the perinatal period: a systematic review. Midwifery. (2019) 76:54–101. 10.1016/j.midw.2019.05.013 31176080

[13] Leach LS Poyser C Cooklin AR Giallo R. Prevalence and course of anxiety disorders (and symptom levels) in men across the perinatal period: a systematic review. J Affect Disord. (2016) 190:675–86. 10.1016/j.jad.2015.09.063 26590515

[14] Vismara L Rollè L Agostini F Sechi C Fenaroli V Molgora S. Perinatal parenting stress, anxiety, and depression outcomes in first-time mothers and fathers: a 3-to 6-months postpartum follow-up study. Front Psychol. (2016) 7:938. 10.3389/fpsyg.2016.00938 27445906 PMC4919353

[15] Pinto TM Figueiredo B Pinheiro LL Canário C. Fathers' parenting self-efficacy during the transition to parenthood. J Reprod Infant Psychol. (2016) 34:343–55. 10.1080/02646838.2016.117885314630529

[16] Parfitt Y Pike A Ayers S. The impact of parents' mental health on parent–baby interaction: a prospective study. Infant Behav Dev. (2013) 36:599–608. 10.1016/j.infbeh.2013.06.003 23850989

[17] Xue X Reed WR Menclova A. Social capital and health: a meta-analysis. J Health Econ. (2020) 72:102317. 10.1016/j.jhealeco.2020.102317 32497954

[18] Ehsan AM De Silva MJ. Social capital and common mental disorder: a systematic review. J Epidemiol Community Health. (2015) 69:1021–8. 10.1136/jech-2015-205868 26179447

[19] Borgonovi F A. Life-cycle approach to the analysis of the relationship between social capital and health in Britain. Soc Sci Med. (2010) 71:1927–34. 10.1016/j.socscimed.2010.09.018 20943301

[20] Lochner K Kawachi I Kennedy BP. Social capital: a guide to its measurement. Health Place. (1999) 5:259–70. 10.1016/S1353-8292(99)00016-7 10984580

[21] Berry HL Welsh JA. Social capital and health in Australia: an overview from the household, income and labor dynamics in Australia survey. Soc Sci Med. (2010) 70:588–96. 10.1016/j.socscimed.2009.10.012 19931236

[22] Fowler K Etchegary H. Economic crisis and social capital: the story of two rural fishing communities. J Occup Organ Psychol. (2008) 81:319–41. 10.1348/096317907X226972

[23] Brehm J Rahn W. Individual-Level Evidence for the Causes and Consequences of Social Capital. Am J Polit Sci. (1997) 1997:999–1023. 10.2307/2111684

[24] Han Y Chung RY-N. Are both individual-level and county-level social capital associated with individual health? a serial cross-sectional analysis in China, 2010–2015. BMJ Open. (2021) 11:e044616. 10.1136/bmjopen-2020-044616 34380714 PMC8359472

[25] Jetten J Haslam SA Cruwys T Greenaway KH Haslam C Steffens NK. Advancing the social identity approach to health and well-being: progressing the social cure research agenda. Eur J Soc Psychol. (2017) 47:789–802. 10.1002/ejsp.233325855820

[26] Cruwys T Dingle GA Haslam C Haslam SA Jetten J Morton TA. Social group memberships protect against future depression, alleviate depression symptoms and prevent depression relapse. Soc Sci Med. (2013) 98:179–86. 10.1016/j.socscimed.2013.09.013 24331897

[27] Kritsotakis G Vassilaki M Melaki V Georgiou V Philalithis AE Bitsios P. Social capital in pregnancy and postpartum depressive symptoms: a prospective mother–child cohort study (the Rhea Study). Int J Nurs Stud. (2013) 50:63–72. 10.1016/j.ijnurstu.2012.08.012 22980484

[28] Nagy E Moore S Silveira PP Meaney MJ Levitan RD Dubé L. Low socioeconomic status, parental stress, depression, and the buffering role of network social capital in mothers. Journal of Mental Health. (2020) 2020:1–8. 10.1080/09638237.2020.1793118 32691647

[29] Eastwood JG Kemp LA Jalaludin BB Phung HN. Neighborhood adversity, ethnic diversity, and weak social cohesion and social networks predict high rates of maternal depressive symptoms: a critical realist ecological study in South Western Sydney, Australia. Int J Health Serv. (2013) 43:241–66. 10.2190/HS.43.2.d 23821904

[30] Ehsan A Klaas HS Bastianen A Spini D. Social capital and health: a systematic review of systematic reviews. SSM-Popul Health. (2019) 8:100425. 10.1016/j.ssmph.2019.100425 31431915 PMC6580321

[31] Firouzbakht M Tirgar A Ebadi A Nia HS Oksanen T Kouvonen A. Psychometric properties of persian version of the short-form workplace social capital questionnaire for female health workers. Int J Occup Environ Med. (2018) 9:184. 10.15171/ijoem.2018.1264 30325359 PMC6466993

[32] Miyajima T Yamaguchi H I. Want to but I Won't: pluralistic ignorance inhibits intentions to take paternity leave in Japan. Front Psychol. (2017) 8:1508. 10.3389/fpsyg.2017.01508 28979216 PMC5611340

[33] Ministry of Health LaW. Basic Survey of Gender Equality in Employment Management 2020 (2021). Available from: https://www.mhlw.go.jp/toukei/list/71-r02.html (accessed 15 December, 2022).

[34] Lofors J. Sundquist K. Low-linking social capital as a predictor of mental disorders: a cohort study of 45 million. Swedes Soc Sci Med. (2007) 64:21–34. 10.1016/j.socscimed.2006.08.024 17011689

[35] Lu W Xiao Y. Adverse childhood experiences and adolescent mental disorders: protective mechanisms of family functioning, social capital, and civic engagement. Health Behav Res. (2019) 2:e1035–e. 10.4148/2572-1836.1035

[36] Zhou C Zheng W Yuan Q Zhang B Chen H Wang W. Associations between social capital and maternal depression: results from a follow-up study in China. BMC Preg Childbirth. (2018) 18:1–9. 10.1186/s12884-018-1673-9 29394914 PMC5797398

[37] Kaneko N Ishigaki K Agawa K. Positive child-rearing feelings of mothers and regional cultural aspect of social capital. J Cult Nurs Stud. (2019) 11:12–21. 10.24658/bunkakango.11.1_1_12

[38] Yagi J Fujiwara T Yambe T Okuyama M Kawachi I Sakai A. Does social capital reduce child behavior problems? Results from the Great East Japan earthquake follow-up for children study. Social Psychiat Psychiatr Epidemiol. (2016) 51:1117–23. 10.1007/s00127-016-1227-2 27168182

[39] Nawa N Isumi A Fujiwara T. Community-level social capital, parental psychological distress, and child physical abuse: a multilevel mediation analysis. Soc Psychiatry Psychiatr Epidemiol. (2018) 53:1221–9. 10.1007/s00127-018-1547-5 29915901

[40] Odagiri Y Ohya Y Inoue S Hayashi T Uchiyama A Takamiya T. Reliablity and validation of the japanese version of the measure of workplace social capital scale (in Japanese). Sangyo Eiseigaku Zasshi. (2010) 83:631.

[41] Kouvonen A Kivimäki M Vahtera J Oksanen T Elovainio M Cox T. Psychometric evaluation of a short measure of social capital at work. BMC Public Health. (2006) 6:1–10. 10.1186/1471-2458-6-251 17038200 PMC1618843

[42] Okano T. Validation and reliability of a Japanese version of the epds. Arch Psychiatric Diag Clin Eval. (1996) 7:523–33.

[43] Nakazato K Mizuguchi K. Studies on psychometric characteristics of depression in the field of internal medicine. Jpn J Psychosomc Med. (1982) 22:107–12.

[44] Rubin DB. Multiple Imputation for Nonresponse in Surveys. New York: Wiley (2004).

[45] Sterne JA White IR Carlin JB Spratt M Royston P Kenward MG. Multiple imputation for missing data in epidemiological and clinical research: potential and pitfalls. BMJ. (2009) 338:157–60. 10.1136/bmj.b2393 19564179 PMC2714692

[46] Takeda T Tsuji Y Kurita H. Psychometric properties of the Japanese Version of the adult attention-deficit hyperactivity disorder (ADHD) self-report scale (ASRS-J) and its short scale in accordance with DSM-5 diagnostic criteria. Res Dev Disabil. (2017) 63:59–66. 10.1016/j.ridd.2017.02.011 28260624

[47] Kurita H Koyama T Osada H. Autism-spectrum quotient-Japanese version and its short forms for screening normally intelligent persons with pervasive developmental disorders. Psychiatry Clin Neurosci. (2005) 59:490–6. 10.1111/j.1440-1819.2005.01403.x 16048456

[48] Yoshida K Yamashita H Conroy S Marks M Kumar C A. Japanese version of mother-to-infant bonding scale: factor structure, longitudinal changes and links with maternal mood during the early postnatal period in Japanese mothers. Arch Womens Ment Health. (2012) 15:343–52. 10.1007/s00737-012-0291-1 22733162 PMC3443344

[49] Fujiwara T Kawachi I A. Prospective study of individual-level social capital and major depression in the United States. J Epidemiol Commun Health. (2008) 62:627–33. 10.1136/jech.2007.064261 18559446

[50] Fujiwara T Yamada F Okuyama M Kamimaki I Shikoro N Barr RG. Effectiveness of educational materials designed to change knowledge and behavior about crying and shaken baby syndrome: a replication of a randomized controlled trial in Japan. Child Abuse Negl. (2012) 36:613–20. 10.1016/j.chiabu.2012.07.003 22954642

[51] Lamarca GA. do C Leal M, Sheiham A, Vettore MV. The association of neighbourhood and individual social capital with consistent self-rated health: a longitudinal study in brazilian pregnant and postpartum women. BMC Preg Childbirth. (2013) 13:1–17. 10.1186/1471-2393-13-1 23324161 PMC3556498

[52] Kouvonen A Oksanen T Vahtera J Stafford M Wilkinson R Schneider J. Low workplace social capital as a predictor of depression: the finnish public sector study. Am J Epidemiol. (2008) 167:1143–51. 10.1093/aje/kwn067 18413361

[53] Woo JM Okusaga O Postolache TT. Seasonality of suicidal behavior. Int J Environ Res Public Health. (2012) 9:531–47. 10.3390/ijerph9020531 22470308 PMC3315262

[54] Sakuraya A Imamura K Inoue A Tsutsumi A Shimazu A Takahashi M. Workplace social capital and the onset of major depressive episode among workers in japan: a 3-year prospective cohort study. J Epidemiol Community Health. (2017) 71:606–12. 10.1136/jech-2016-208561 28235820

[55] Kawachi I Kennedy BP Glass R. Social capital and self-rated health: a contextual analysis. Am J Public Health. (1999) 89:1187–93. 10.2105/AJPH.89.8.1187 10432904 PMC1508687

[56] Harnois G Gabriel P. Mental Health and Work: Impact, Issues and Good Practices. (2000). New York: Cornell University.

[57] Firouzbakht M Tirgar A Oksanen T Kawachi I Hajian-Tilaki K Nikpour M. Workplace social capital and mental health: a cross-sectional study among iranian workers. BMC Public Health. (2018) 18:794. 10.1186/s12889-018-5659-3 29940919 PMC6019288

[58] Gao J Weaver SR Dai J Jia Y Liu X Jin K. Workplace social capital and mental health among chinese employees: a multi-level, cross-sectional study. PLoS ONE. (2014) 9:e85005. 10.1371/journal.pone.0085005 24404199 PMC3880334

[59] Ando S Nishida A Yamasaki S Koike S Morimoto Y Hoshino A. Cohort profile: the Tokyo teen cohort study (Ttc). Int J Epidemiol. (2019) 48:1414–g. 10.1093/ije/dyz033 30879075 PMC6857749

[60] Mao Q Zhu LX Su XY. A comparison of postnatal depression and related factors between Chinese new mothers and fathers. J Clin Nurs. (2011) 20:645–52. 10.1111/j.1365-2702.2010.03542.x 21320193

[61] Sommer TE Sabol TJ Chase-Lansdale PL Small M Wilde H Brown S. Promoting parents' social capital to increase children's attendance in head start: evidence from an experimental intervention. J Res Educ Eff. (2017) 10:732–66. 10.1080/19345747.2016.1258099

[62] Fielden JM Gallagher LM. Building social capital in first-time parents through a group-parenting program: a questionnaire survey. Int J Nurs Stud. (2008) 45:406–17. 10.1016/j.ijnurstu.2006.09.008 17097090

