# Supplementary material for: Association of Paternal Workplace and Community Social Capital With Paternal Postnatal Depression and Anxiety: A Prospective Study
Source: Front Psychiatry. 2022 Feb 17;13:782939. doi: 10.3389/fpsyt.2022.782939 (PMC8892241; doi:10.3389/fpsyt.2022.782939)
Supplement: Supplementary file 1 [file Data_Sheet_1.docx]

| Supplemental Table 1 Correlation of paternal social network | | | |
| --- | --- | --- | --- |
|  | 1 | 2 | 3 |
| 1. Workplace social capital |  |  |  |
| 2. Community social capital | 0.17^**^ |  |  |
| 3. Social participation in the last year before delivery | 0.08 | −0.13^*^ |  |
| 4. Number of people who consult about parenting | 0.19^**^ | 0.13^*^ | −0.10 |
| SD = standard deviation |  |  |  |
| **p<0.01, *<0.05 |  |  |  |

| Supplemental Table 2 Association between paternal social capital and paternal depressive symptoms at 3 months before delivery before multiple imputation | | | | | | | | | |
| --- | --- | --- | --- | --- | --- | --- | --- | --- | --- |
|  |  | Workplace social capital | | | Community social capital | | |  |  |
|  |  | Crude | Model 1 | Model 2 | Crude | Model 1 | Model 2 | Model 3 |  |
|  |  | β (95%CI) | β (95%CI) | β (95%CI) | β (95%CI) | β (95%CI) | β (95%CI) | β (95%CI) |  |
| Workplace social capital |  | **−0.69 (−1.16 to −0.22)** | **−0.58 (−1.13 to −0.03)** | −0.15 (−0.63 to 0.34) |  |  |  | 0.12 (−0.39 to 0.64) |  |
| Community social capital |  |  |  |  | **−0.25 (−0.38 to −0.12)** | **−0.30 (−0.45 to −0.15)** | **−0.28 (−0.41 to −0.15)** | **−0.29 (−0.43 to −0.15)** |  |
| Obstetrics hospital | A |  | Ref | Ref |  | Ref | Ref | Ref |  |
|  | B |  | −0.43 (−1.40 to 0.55) | 0.11 (−0.74 to 0.96) |  | −0.42 (−1.38 to 0.54) | 0.12 (−0.71 to 0.94) | 0.29 (−0.58 to 1.17) |  |
| Paternal age |  |  | −0.05 (−0.14 to 0.03) | −0.05 (−0.12 to 0.03) |  | −0.04 (−0.12 to 0.05) | −0.03 (−0.10 to 0.04) | −0.03 (−0.11 to 0.04) |  |
| Paternal education | High school or less |  | 0.01 (−1.57 to 1.60) | 0.04 (−1.34 to 1.42) |  | 0.23 (−1.32 to 1.78) | 0.12 (−1.21 to 1.45) | 0.06 (−1.34 to 1.46) |  |
|  | Some college |  | −1.34 (−2.95 to 0.27) | −1.28 (−2.67 to 0.12) |  | **−1.72 (−3.31 to −0.12)** | **−1.66 (−3.02 to −0.29)** | **−1.79 (−3.21 to −0.37)** |  |
|  | College or more |  | Ref | Ref |  | Ref | Ref | Ref |  |
|  | Unknown |  | NA | NA |  | NA | NA | NA |  |
| Annual household income (JPN yen) | ≦2,000,000 |  | NA | NA |  | NA | NA | NA |  |
|  | 2,010,000 - 4,000,000 |  | 1.14 (−0.67 to 2.95) | 0.69 (−0.88 to 2.26) |  | 1.14 (−0.63 to 2.91) | 0.63 (−0.89 to 2.15) | 0.78 (−0.81 to 2.38) |  |
|  | 4,010,000 - 6,000,000 |  | 1.09 (−0.36 to 2.53) | 0.69 (−0.57 to 1.94) |  | 1.31 (−0.11 to 2.73) | 0.89 (−0.33 to 2.11) | 1.03 (−0.27 to 2.34) |  |
|  | 6,010,000 - 8,000,000 |  | Ref | Ref |  | Ref | Ref | Ref |  |
|  | 8,010,000 - 10,000,000 |  | 1.08 (−0.30 to 2.45) | **1.39 (0.19 to 2.60)** |  | 1.31 (−0.05 to 2.67) | **1.68 (0.51 to 2.85)** | **1.67 (0.44 to 2.89)** |  |
|  | 10,000,000 - 15,000,000 |  | **1.39 (0.10 to 2.68)** | **1.54 (0.42 to 2.66)** |  | **1.32 (0.06 to 2.57)** | **1.58 (0.51 to 2.66)** | **1.65 (0.50 to 2.81)** |  |
|  | 15,001,000+ |  | 0.34 (−1.36 to 2.04) | 0.72 (−0.77 to 2.21) |  | 0.30 (−1.37 to 1.97) | 0.69 (−0.76 to 2.13) | 0.79 (−0.70 to 2.28) |  |
| Paternal ACE total score | 0 |  | Ref | Ref |  | Ref | Ref | Ref |  |
|  | 1 |  | 0.23 (−0.97 to 1.42) | 0.22 (−0.83 to 1.26) |  | 0.13 (−1.04 to 1.31) | 0.14 (−0.28 to 1.35) | 0.22 (−0.85 to 1.29) |  |
|  | 2+ |  | 1.60 (−0.64 to 3.84) | 0.95 (−0.99 to 2.90) |  | 1.40 (−0.80 to 3.60) | −0.17 (−1.14 to 0.80) | 0.35 (−1.66 to 2.36) |  |
| Paternal employment | Full-time job |  | Ref | Ref |  | Ref | Ref | Ref |  |
|  | Part-time job |  | 4.20 (−1.19 to 9.60) | 1.95 (−2.78 to 6.69) |  | 3.10 (−2.22 to 8.42) | 1.10 (−3.50 to 5.70) | 1.00 (−0.72 to 5.72) |  |
|  | Self-employed |  | 1.65 (−2.22 to 5.51) | 0.23 (−3.15 to 3.61) |  | 1.40 (−2.39 to 5.18) | 0.29 (−2.98 to 3.55) | 0.03 (−3.32 to 3.38) |  |
|  | Others |  | −0.51 (−6.07 to 5.05) | 1.73 (−3.12 to 6.58) |  | −0.11 (−5.57 to 5.35) | 2.25 (−2.46 to 6.96) | 3.92 (−3.22 to 11.06) |  |
| Paternal childcare leave | Yes or Planning to take |  | Ref | Ref |  | Ref | Ref | Ref |  |
|  | No |  | −0.74 (−1.99 to 0.52) | −0.51 (−1.61 to 0.59) |  | −1.01 (−2.23 to 0.22) | −0.67 (−1.73 to 0.39) | −0.75 (−1.88 to 0.37) |  |
| Paternal history of psychiatric disorders | No |  | Ref | Ref |  | Ref | Ref | Ref |  |
|  | Yes |  | 1.17 (−2.17 to 4.50) | −1.53 (−4.24 to 1.19) |  | 1.36 (−1.89 to 4.61) | −１.84 (−4.72 to 1.05) | −1.99 (−5.06 to 1.08) |  |
| History of delivery | First birth |  | Ref | Ref |  | Ref | Ref | Ref |  |
|  | Multiparity |  | −0.08 (−1.01 to 0.84) | 0.23 (−0.60 to 1.06) |  | 0.28 (−0.65 to 1.21) | 0.53 (−0.28 to 1.35) | 0.47 (−0.39 to 1.34) |  |
| Normal pregnancy | Yes |  | Ref | Ref |  | Ref | Ref | Ref |  |
|  | No |  | −0.17 (−1.32 to 0.98) | −0.14 (−1.14 to 0.86) |  | −0.18 (−1.30 to 0.95) | −0.17 (−1.15 to 2.64) | −0.20 (−1.21 to 0.81) |  |
| Paternal feelings when pregnancy was confirmed | Happy |  | Ref | Ref |  | Ref | Ref | Ref |  |
|  | Unexpected but happy/unexpected and confused/did not know what to do/no feelings/other |  | 0.84 (−0.36 to 2.03) | 0.28 (−0.76 to 1.33) |  | 0.76 (−0.41 to 1.93) | 0.17 (−0.85 to 1.18) | 0.18 (−0.90 to 1.26) |  |
| Paternal depressive symptoms at 1 week after delivery |  |  |  | **0.68 (0.55 to 0.82)** |  |  | **0.68 (0.55 to 0.81)** | **0.68 (0.54 to 0.82)** |  |
| Maternal depressive symptoms at 1 week after delivery |  |  |  | 0.06 (−0.07 to 0.19) |  |  | 0.05 (−0.08 to 0.17) | 0.05 (−0.09 to 0.18) |  |
| Maternal anxiety at 1 week after delivery |  |  |  | −0.02 (−0.07 to 0.04) |  |  | −0.02 (−0.07 to 0.03) | −0.02 (−0.07 to 0.04) |  |
| Number of people who can consult about parenting |  |  |  |  |  |  |  | −0.02 (−0.08 to 0.03) |  |
| 95%CI=95% Confidence Interval. | | | | | | | | | |
| Model 1 adjusted paternal education, paternal age, annual household income, paternal employment, paternal childcare leave, paternal adverse childhood experiences, paternal history of psychiatric disorder, history of delivery, obstetrics hospital, normal pregnancy, and paternal feeling when pregnancy was confirmed. | | | | | | | | | |
| Model 2 added paternal depressive symptoms at 1 week after delivery, maternal depressive symptoms and anxiety at 1 week after delivery into Model 1. | | | | | | | | | |
| Model 3 included number of people who can consult about parenting and both types of social capital into Model 2. | | | | |  |  |  |  |  |

| Supplemental Table 3 Association between paternal social capital and paternal anxiety at 3 months after delivery before multiple imputation | | | | | | | | |
| --- | --- | --- | --- | --- | --- | --- | --- | --- |
|  |  | Workplace social capital | | | Community social capital | | |  |
|  |  | Crude | Model 1 | Model 2 | Crude | Model 1 | Model 2 | Model 3 |
|  |  | β (95%CI) | β (95%CI) | β (95%CI) | β (95%CI) | β (95%CI) | β (95%CI) | β (95%CI) |
| Workplace social capital |  | **−3.07 (−4.14 to −2.00)** | **−2.83 (−4.10 to −1.57)** | **−0.71 (−1.81 to −0.39)** |  |  |  | −0.44 (−1.59 to 0.72) |
| Community social capital |  |  |  |  | **−0.49 (−0.80 to −0.17)** | **−0.64 (−1.00 to −0.29)** | **−0.46 (−0.74 to −0.17)** | **−0.42 (−0.72 to −0.12)** |
| Obstetrics hospital | A |  | Ref | Ref |  | Ref | Ref | Ref |
|  | B |  | −2.06 (−4.30 to 0.17) | −0.57 (−2.43 to 1.29) |  | −2.06 (−4.33 to 0.19) | −0.55 (−2.38 to 1.29) | −0.16 (−2.04 to 1.72) |
| Paternal age |  |  | 0.03 (−0.17 to 0.22) | −0.01 (−0.17 to 0.15) |  | 0.07 (−0.13 to 0.27) | 0.01 (−0.49 to 0.17) | 0.003 (−0.16 to 0.17) |
| Paternal education | High school or less |  | −1.25 (−4.89 to 2.40) | −0.06 (−3.06 to 2.95) |  | −0.36 (−4.03 to 3.31) | 0.20 (−2.75 to 3.15) | 0.41 (−2.60 to 3.42) |
|  | Some college |  | −1.29 (−5.00 to 2.41) | −0.74 (−3.78 to 2.30) |  | −2.02 (−5.79 to 1.76) | −1.32 (−4.34 to 1.71) | −1.98 (−5.03 to 1.06) |
|  | College or more |  | Ref | Ref |  | Ref | Ref | Ref |
|  | Unknown |  | NA | NA |  | NA | NA | NA |
| Annual household income (JPN yen) | ≦2,000,000 |  | NA | NA |  | NA | NA | NA |
|  | 2,010,000 - 4,000,000 |  | 0.50 (−3.65 to 4.65) | −0.86 (−4.28 to 2.95) |  | 0.75 (−3.44 to 4.94) | −0.91 (−4.28 to 2.46) | −1.10 (−4.52 to 2.33) |
|  | 4,010,000 - 6,000,000 |  | 3.06 (−0.25 to 6.38) | **3.20 (0.48 to 5.92)** |  | **3.63 (0.27 to 6.99)** | **3.56 (0.87 to 6.26)** | **3.87 (1.08 to 6.66)** |
|  | 6,010,000 - 8,000,000 |  | Ref | Ref |  | Ref | Ref | Ref |
|  | 8,010,000 - 10,000,000 |  | 2.34 (−0.82 to 5.50) | 1.29 (−1.33 to 3.92) |  | 2.44 (−0.77 to 5.65) | 1.62 (−0.98 to 4.22) | 2.12 (−0.52 to 4.74) |
|  | 10,000,000 - 15,000,000 |  | 1.83 (−1.14 to 4.79) | 1.71 (−0.73 to 4.15) |  | 1.17 (−1.81 to 4.14) | 1.64 (−0.74 to 4.03) | 2.11 (−0.37 to 4.58) |
|  | 15,001,000+ |  | 0.79 (−3.12 to 4.69) | 1.10 (−2.14 to 4.33) |  | 0.53 (−3.42 to 4.74) | 0.99 (−2.20 to 4.18) | 1.54 (−1.66 to 4.74) |
| Paternal ACE total score | 0 |  | Ref | Ref |  | Ref | Ref | Ref |
|  | 1 |  | 0.01 (−2.74 to 2.75) | 0.44 (−1.84 to 2.72) |  | −0.15 (−2.93 to 2.63) | 0.33 (−1.92 to 2.58) | 0.05 (−2.25 to 2.35) |
|  | 2+ |  | 3.44 (−1.70 to 8.59) | −1.21 (−5.51 to 3.09) |  | 3.17 (−2.04 to 8.38) | −1.55 (−5.80 to 2.70) | −0.46 (−4.81 to 3.89) |
| Paternal employment | Full-time job |  | Ref | Ref |  | Ref | Ref | Ref |
|  | Part-time job |  | 2.56 (−9.83 to 14.96) | −2.17 (−12.46 to 8.12) |  | −0.16 (−12.74 to 12.43) | −3.69 (−13.86 to 6.48) | −3.10 (−13.20 to 6.99) |
|  | Self-employed |  | 0.83 (−8.05 to 9.72) | −1.19 (−8.54 to 6.16) |  | −0.56 (−9.52 to 8.40) | −1.34 (−8.57 to 5.89) | −1.76 (−8.93 to 5.42) |
|  | Others |  | −0.60 (−13.36 to 12.17) | 3.26 (−13.80 to 7.28) |  | −0.43 (−13.35 to 12.49) | −2.66 (−13.06 to 7.74) | −0.69 (−15.92 to 14.55) |
| Paternal childcare leave | Yes or Planning to take |  | Ref | Ref |  | Ref | Ref | Ref |
|  | No |  | −1.22 (−4.10 to 1.66) | −1.46 (−3.85 to 0.93) |  | −2.18 (−5.08 to 0.72) | −1.84 (−4.19 to 0.50) | −1.60 (−4.01 to 0.82) |
| Paternal history of psychiatric disorders | No |  | Ref | Ref |  | Ref | Ref | Ref |
|  | Yes |  | −2.18 (−9.84 to 5.49) | −1.50 (−7.93 to 4.93) |  | −0.48 (−8.17 to 7.21) | −1.18 (−7.45 to 5.10) | −1.48 (−7.96 to 5.00) |
| History of delivery | First birth |  | Ref | Ref |  | Ref | Ref | Ref |
|  | Multiparity |  | 0.30 (−1.83 to 2.52) | −0.05 (−1.86 to 1.77) |  | 1.05 (−1.15 to 3.24) | 0.42 (−1.39 to 2.24) | −0.24 (−2.11 to 1.62) |
| Normal pregnancy | Yes |  | Ref | Ref |  | Ref | Ref | Ref |
|  | No |  | 0.90 (−1.73 to 3.54) | 0.76 (−1.42 to 2.95) |  | 1.08 (−1.58 to 3.74) | 0.77 (−1.38 to 2.92) | 0.35 (−1.83 to 2.52) |
| Paternal feelings when pregnancy was confirmed | Happy |  | Ref | Ref |  | Ref | Ref | Ref |
|  | Unexpected but happy/unexpected and confused/did not know what to do/no feelings/other |  | 1.28 (−1.46 to 4.01) | 0.02 (−2.24 to 2.29) |  | 1.37 (−1.40 to 4.14) | −0.10 (−2.34 to 2.14) | 0.24 (−2.07 to 2.55) |
| Paternal anxiety at 1 week after delivery |  |  |  | **0.63 (0.53 to 0.73)** |  |  | **0.64 (0.54 to 0.74)** | **0.63 (0.53 to 0.74)** |
| Maternal depressive symptoms at 1 week after delivery |  |  |  | 0.11 (−0.18 to 0.39) |  |  | 0.07 (−0.21 to 0.35) | 0.13 (−0.16 to 0.42) |
| Maternal anxiety at 1 week after delivery |  |  |  | −0.01 (−0.12 to 0.10) |  |  | −0.01 (−0.12 to 0.10) | −0.01 (−0.12 to 0.11) |
| Number of people who can consult about parenting |  |  |  |  |  |  |  | 0.004 (−0.11 to 0.11) |
| 95%CI=95% Confidence Interval. | | | | | | | | |
| Model 1 adjusted paternal education, paternal age, annual household income, paternal employment, paternal childcare leave, paternal adverse childhood experiences, paternal history of psychiatric disorder, history of delivery, obstetrics hospital, normal pregnancy, and paternal feeling when pregnancy was confirmed. | | | | | | | | |
| Model 2 added paternal anxiety at 1 week after delivery, maternal depressive symptoms and anxiety at 1 week after delivery into Model 1. | | | | | | | | |
| Model 3 included number of people who can consult about parenting and both types of social capital into Model 2. | | | | |  |  |  |  |
